# Analysis of *Bordetella pertussis *pertactin and pertussis toxin types from Queensland, Australia, 1999–2003

**DOI:** 10.1186/1471-2334-6-53

**Published:** 2006-03-16

**Authors:** Shane Byrne, Andrew T Slack

**Affiliations:** 1 Public Health Microbiology, Queensland Health Scientific Services, Brisbane, Australia; 2 Molecular Pathology Department, Sullivan Nicolaides Pathology, Brisbane, Australia

## Abstract

**Background:**

In Australia two acellular *Bordetella pertussis *vaccines have replaced the use of a whole cell vaccine. Both of the licensed acellular vaccines contain the following three components; pertussis toxoid, pertussis filamentous haemagglutinin and the 69 kDa pertactin adhesin. One vaccine also contains pertussis fimbriae 2 and 3. Various researchers have postulated that herd immunity due to high levels of pertussis vaccination might be influencing the makeup of endemic *B. pertussis *populations by selective pressure for strains possessing variants of these genes, in particular the pertactin gene type. Some publications have suggested that *B. pertussis *variants may be contributing to a reduced efficacy of the existing vaccines and a concomitant re-emergence of pertussis within vaccinated populations. This study was conducted to survey the pertactin and pertussis toxin subunit 1 types from *B. pertussis *isolates in Queensland, Australia following the introduction of acellular vaccines.

**Methods:**

Forty-six *B. pertussis *isolates recovered from Queensland patients between 1999 and 2003 were examined by both DNA sequencing and LightCycler™ real time PCR to determine their pertactin and pertussis toxin subunit 1 genotypes.

**Results:**

Pertactin typing showed that 38 isolates possessed the *prn*1 allele, 3 possessed the *prn2 *allele and 5 possessed the *prn3 *allele. All forty-six isolates possessed the pertussis toxin *ptxS1A *genotype. Amongst the circulating *B. pertussis *population in Queensland, 82.5% of the recovered clinical isolates therefore possessed the *prn1*/*ptxS1A *genotype.

**Conclusion:**

The results of this study compared to historical research on Queensland isolates suggest that *B. pertussis *pertactin and pertussis toxin variants are not becoming more prevalent in Queensland since the introduction of the acellular vaccines. Current prevalences of pertactin variants are significantly different to that described in a number of other countries with high vaccine coverage. Relative paucity of recovered isolates compared to notified infections, due primarily to non culture based pertussis diagnostics is however a confounding factor in the assessment of variant prevalence.

## Background

*Bordetella pertussis*, the etiological agent of 'Whooping Cough' remains prevalent in Australia despite the introduction and wide spread use of pertussis vaccines as part of the childhood immunisation scheme. The Australian standard vaccination schedule for pertussis consists of acellular vaccines given in doses at 2, 4 and 6 months, followed by a fourth dose at 4 years and a booster at 15–17 years of age [1]. Prior to 1999 a local whole cell vaccine was in use beginning in the decade 1936–1945. [2]. An 'Immunise Australia' program established in 1997 has set a target of 90% coverage for pertussis vaccination [2]. In the Australian state of Queensland pertussis vaccine coverage in the 1990s moved from the high 70% to mid 80%, and then rose above the 90% target from 2001 onwards [2-4]. In spite of this high vaccine coverage, in recent times pertussis infection has been the most common vaccine preventable illness in Australia, with epidemics occurring each 3 to 5 years associated with a background of endemic circulation [5]. Pertussis notifications per 100,000 of the Australian population have risen from 4.9 in 1992 to an average of 34.3 (range: 25.6 to 84.5) through to 2004 [6]. In Queensland, pertussis notifications throughout the 1990s and into the new decade align closely with the surge evident in the national figures [6]. Many other countries with high vaccination coverage similar to that in Australia have also seen a resurgence of pertussis disease [7-10]. A number of factors have been postulated for explaining this global resurgence of pertussis disease within highly vaccinated communities.

Presently the primary cause is thought to be increased cases in adults and adolescents due to waning immunity, resulting in a reservoir of infection for non or incompletely vaccinated young children [8,11,12]. Australian pertussis infection data appears to support this observation, whereby from 1991 to 2002 approximately 60% of Australian pertussis notifications occurred in people over 10 years of age [2-4,13]. To address this situation the 'Global Pertussis Initiative' has recommended booster immunisation for older children and adolescents [14,15]. This recommendation has been taken up in Australia from 2004 with the licensing, funding and inclusion of the GlaxoSmithKline acellular booster 'Boostrix' in the standard vaccination schedule for 15–17 year olds [1,16]. Underpinning the increase in adult/adolescent notifications has also been significant improvement in the recognition of pertussis infections in this group through improved surveillance and the introduction of new testing methods such as nucleic acid amplification (NAA) and improved serological diagnosis. Improved immunoassays targeting both the *B. pertussis *antigen (IgA) and specifically in Queensland an anti-pertussis toxin IgG have both contributed to increased detection and notifications [13,17,18].

A second hypothesis for explaining the re-emergence of pertussis disease within highly vaccinated communities is vaccination-induced *B. pertussis *evolution, originally tendered in the literature by Mooi et.al [19]. This hypothesis involves a process whereby herd immunity drives the emergence of strains possessing variant genes and a resultant expression of proteins which are a mismatch with existing vaccine components. A particular concern based on this hypothesis is the impact on vaccine efficacies in light of the trend towards replacement of whole cell vaccines (WCV) with acellular vaccines (ACV) containing only a few select immunogenic antigens. As ACVs contain only selected *B. pertussis *antigens such as pertussis toxoid (PT), pertussis filamentous haemaglutinin (FHA) and pertactin (PRN), it has been postulated that high levels of vaccination coverage places selective pressure on the genes of these vaccine targets. In the first publication suggesting possible vaccination driven immune evasion adaptations were occurring in *B. pertussis*, Mooi and co-workers described a situation in the Netherlands where prior to the introduction of a WCV the endemic PRN type was solely *prn1*. Post WCV introduction the *prn1 *frequency dropped to approximately 10% and the remaining majority were found to belong to the non-vaccine types *prn2 *and *prn3 *[9,19]. Since then further evidence supporting this hypothesis has been noted with significant replacement of vaccine type PRN and PT alleles in the endemic *B. pertussis *populations in a number of other European and American countries, in particular replacement of vaccine type *prn1 *and *ptxS1B *with variants [11].

Whilst a shift in the PRN and PT types has certainly occurred in a large number of countries, recent work has suggested that this shift may be independent of the use of vaccines. Firstly, like continental Europe *ptxS1A *has also become predominant in the UK despite the fact that a highly effective UK WCV actually contains a *ptxS1A *derived protein [20]. Also, recent work utilising a mouse model has shown that ACVs do in fact remain efficacious against a group of strains which possess variant alleles, provided that the ACV is of sufficient potency [21]. Further more, Japan which has the worlds longest standing ACV based pertussis vaccination program has not shown significant emergence, let alone dominance of pertussis strains possessing PRN or PT differing to the ACVs in use [22,23]. Recent population profiling of *B. pertussis *in five European countries has also shown no correlation between strain characteristics and the use of differing vaccination programs [24]. And a majority of isolates from those same five European countries also share the same PFGE profile despite differences in vaccination policies and vaccine types and contents [25]. Evasion of Bordetella bacteriophage infection has recently been mooted as a possible alternate explanation for the observed PRN variability [11]. A recent review of isolate polymorphism and vaccination programs in relation to pertussis re-emergence concluded that to date no direct link has been noted for PRN and PT variation [11]. The authors do state however that it is 'possible but not yet proven' that the emergence of variants coupled to waning immunity may have resulted in the resurgence of pertussis in the older children and adolescents group, but reiterate that reduced circulation and waning immunity may also have produced a similar outcome [11].

**Figure 1:**
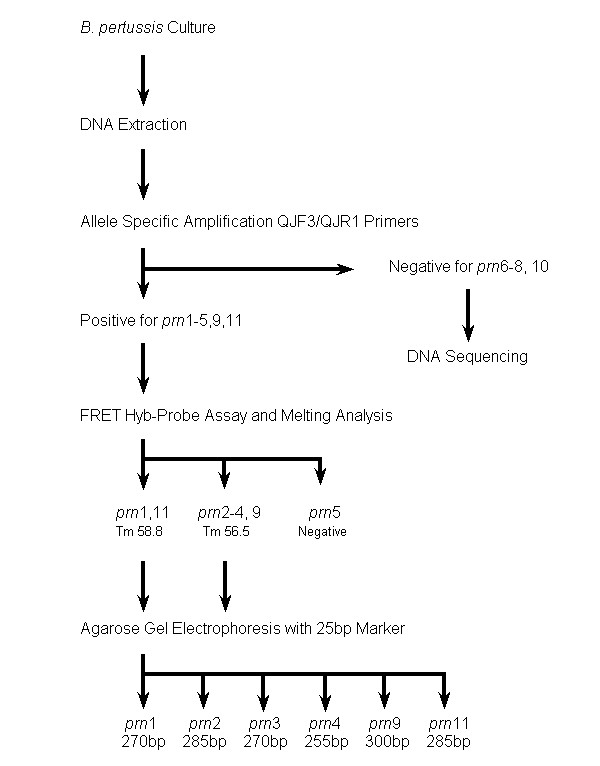
**Flowchart Depicting Modified Protocols for LightCycler Based *prn *Allelic Typing**. Modified flowchart of protocols from Mäkinen et al. detailing the updated procedure for LightCycler based allelic typing of the *B. pertussis prn *gene. Electrophoretic sizing of all QH8F-QH2R PCR products from the FRET HybProbe assay in addition to melting curve analysis enables determination of new alleles detected since publication of the original method.

PRN is a 69-kDa outer membrane protein that mediates adherence of the bacterium to host cell walls. The majority of the polymorphism in PRN occurs within a repetitive element of the gene which contains a variable number of Gly-Gly-X-X-Pro (GGXXP) repeats [19]. Currently eleven different PRN allelic types have been discovered through the use of DNA sequencing [26]. Various methods for the identification of PRN alleles have been used including full or partial gene sequencing, shared primer PCR product size polymorphism, amplification-refractory mutation system PCR and real time PCR with probes or DNA binding dyes including product melting curve analysis and electrophoretic sizing [19,20,26,27]. Full gene DNA sequencing produces the highest fidelity information on emerging PRN variants, and uniquely so for those with mutations outside of the GGXXP region. However, the size of the PRN gene renders full gene sequencing a time consuming and moderately costly exercise for allelic typing. Mäkinen et al. describe two real time PCR assays, that when used within a scheme specifically identify the majority of PRN alleles [28]. We have utilised a corrected adaptation of the Mäkinen et al. scheme (represented in Figure 1), whereby electrophoretic sizing of all HybProbe PCR reaction products irrespective of the product melting point values updates the scheme enabling specific discrimination of the new alleles detected since publication of the article.

The PT protein is a typical AB exotoxin consisting of five subunits of which the 269 amino acid S1 subunit encodes the active toxic function [29,30]. Four polymorphisms have currently been described for the PT S1 subunit. Commonly strains used in vaccines have either the *ptxS1B *or *ptxS1D *alleles. The detection of PT alleles has until recently also been performed exclusively by DNA sequencing, however a paper has been recently published utilising real-time PCR and product melting curve analysis for the differentiation of the currently known PT alleles [31].

In Australia, two acellular *B. pertussis *vaccines which were licensed and made available from 1997 have totally replaced the use of a locally manufactured whole cell vaccine since 1999. Both of the licensed ACVs contain the following three components; PT, FHA and PRN. One of the vaccines also contains pertussis fimbriae 2 and 3. The Australian WCV in use until 1999 was manufactured from a strain possessing *prn1 *and *ptxS1A *[32]. The GlaxoSmithKline (GSK) acellular vaccine is derived from a strain possessing *prn1*/*ptxS1B*, the manufacturer of the other licensed acellular vaccine declined to provide details of the specific alleles contained in their preparation. This aim of this study was to investigate circulating PRN and PT alleles in Queensland *B. pertussis *isolates post introduction of ACVs. In total, forty-six clinical isolates collected from 1999 to 2003 were available. Publication of data from 1989–1999 including an earlier collection of Queensland isolates by Poynten et al. [32] whilst this work was in progress has also enabled comparisons to be drawn between the prevalences of alleles between the two time periods of 1989–1999 and 1999–2003.

## Methods

### Bacterial strains and culture conditions

A total of forty-six unique clinical isolates of *B. pertussis *collected and stored from 1999 through 2003 were retrieved from the frozen culture collections of Public Health Microbiology, Queensland Health Scientific Services and the Microbiology Department of Sullivan Nicolaides Pathology, Taringa, Brisbane. Frozen isolates were recovered and grown on Charcoal Agar (Biomerieux Australia) incubated at 35°C for four days in moist conditions. Demographic data including patient name, date of birth, postal code and date of isolation was available for all strains, but there was very limited information on patient vaccination history (3 of 46 patients).

### Extraction of DNA

Genomic DNA from the *B. pertussis *isolates was extracted using the High Pure PCR Template Preparation Kit (Roche Diagnostics) according to the manufacturers instructions.

### PRN and PT typing

Examination of the PRN and PT alleles carried by the isolates was done by DNA sequencing utilising a combination of the methods of Mooi et al. [19,33], and the expansion of those methods by Fry et al. [20]. Two overlapping amplicons of the pertactin gene were generated from each DNA extract utilising the primer pairs PR8F/PRN1618 and BF/PR5R [20]. PCR reactions were performed in 50 μL final volumes comprising 1× GeneAmp PCR Buffer II (Applied Biosystems), 1.5 mM MgCl_2 _(Applied Biosystems), 200 μM deoxynucleotides (Amersham Biosciences), 10 pmol of each primer (Geneworks, Adelaide, Australia), 1.5 Units of AmpliTaq Gold DNA Polymerase (Applied Biosystems), 10% DMSO (D2438, Sigma Chemical Co., St Louis) and the remainder up to 48 μL as Water for Injections BP (AstraZeneca). 2 μL of template DNA was added to complete the reaction mix. Thermal cycling was performed using a GeneAmp PCR System 9700 thermal cycler (Applied Biosystems) with the following conditions: initial denaturation 95°C for 9 minutes, thirty-five cycles consisting of 95°C for 30 seconds, 57°C for 30 seconds and 72°C for 1 minute, a single final extension of 72°C for 7 minutes and a hold at 4°C. Sequencing of each of the two overlapping sub-fragments of the *prn *gene amplified from each isolate was performed with the primers used to generate the PCR product as well as those remaining primers used by Fry et al. [20] which were located internal to the respective fragment. Cycle sequencing was performed using the Big Dye Terminator Cycle Sequencing Ready Reaction Kit V1.1 (Applied Biosystems) according to manufacturers instructions. Cycle sequencing reaction products were purified using sodium acetate/ethanol precipitation, and subsequently analysed on an ABI PRISM 377 DNA Sequencer (Applied Biosystems). Sequences were assembled and analysed using Sequencher 3.0 software (Gene Codes Corporation). The primer pair S1F/S1R of Mooi et al. [19] were utilised to amplify the polymorphic region of the PT subunit 1 gene. The reaction was performed using identical master-mix and cycling conditions as detailed for the PRN typing PCR, substituting the S1F/S1R primers. The PT subunit 1 PCR fragments were sequenced also as above using the three primers S1F, S1R and S1FM [19].

**Figure 2:**
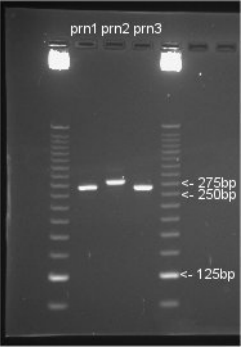
**QH8F-QH2R PCR Product Sizing**. Ethidium bromide stained 3% agarose gel electrophoresis of QH8F-QH2R primer pair PCR products for sequence confirmed *prn1*, *prn2 *and *prn3 *containing *B. pertussis *isolates. Sizing against Invitrogen 25 bp DNA Ladder illustrates PCR product sizes of 270 bp, 285 bp and 270 bp respectively.

In addition to sequencing, a corrected and modified PRN typing scheme originally developed by Mäkinen et al. [28] utilising real time PCR was also applied to investigate the applicability of this rapid method for Australian isolates. Allele specific amplification (ASA) was first performed and samples that were positive were then analysed using a Förster Resonance Energy Transfer (FRET) hybridisation probe assay. Both procedures were performed as described by Mäkinen et al. [28], except that total cycles were increased to 40 and LightCycler FastStart DNA Master kits (Roche Diagnostics) were utilised requiring a 10 minute pre-incubation at 95°C to accommodate the activation of the FastStart enzyme. Melting curve analysis of products was performed, followed by post amplification recovery of all PCR products from the capillaries by inversion centrifugation. Agarose gel electrophoresis using 3% agarose and a 25 bp DNA ladder (Invitrogen) was then performed for the determination of PCR product size. Electrophoretic sizing of all PCR products is a required modification of the original methods in order to successfully use the Mäkinen et al. [28] scheme with currently known PRN alleles. In addition we found that the PCR product sizes for all PRN types described in the text of Mäkinen et al. [28] are in fact 10 base pairs understated. This observation was confirmed by software PCR product size prediction based on the binding sites of primers QH8F and QH2R within the PRN variant gene sequences stored in Genbank, and by experimental observations of PRN types in this study (Figure 2). In light of the discovery of new PRN variants 9 and 11, it now becomes paramount to electrophoretically size every positive QH8F/QH2R HybProbe assay PCR product, preferably with a 25 bp graduated DNA marker. Since *prn11 *is identical under the HybProbes QJ1/QJ2 to *prn1*, it is predicted to have the same melting temperature (Tm) value, however the size of the PCR products differ with *prn1 *being 270 bp, and *prn11 *285 bp. Also, as *prn9 *is identical under the HybProbes to *prn2*-*4*, it would likewise be predicted to have an identical Tm value to those alleles, however the PCR product size of 300 bp clearly differentiates *prn9 *from *prn2*-*4*.

### Statistical analysis

A two tailed Fishers exact test was utilised to test for statistically significant differences between the observed and historical proportions of the PRN alleles. The two-tailed P value was calculated by the recommended method of summing small P values. Statistical analysis was performed utilising the online calculators of GraphPad Software. At P values <0.05 the distributions of the alleles cannot be said to be significantly different due to chance [34]. Sample size calculations were performed using the online calculators of Raosoft, Inc.

## Results and discussion

### Age and sex distribution

A slightly higher rate of infection of females over males of 1.3:1 (Table 1) was evident amongst the patients from which the isolates were collected and is comparable to that previously described in reviews of Australian pertussis notification data [2-4]. The age distribution of patients from this collection was <1 years (n = 22), 1–4 years (n = 8), 5–9 years (n = 1), 10–14 years (n = 3) and >15 years (n = 12). This age distribution agrees with previous reports which indicate infants who may be only partly or not-vaccinated have the greatest disease burden [35]. It also reflects the observation of a re-emergence of pertussis in the adolescent and adult demographic which has been reported as the major reservoir for transmission to infants [15].

**Table 1:** Queensland *Bordetella pertussis *Sample Petactin and Pertussis Toxin Alleles 1999–2003

Year	Number of PRN Types			PT (All *ptxS1*A)	Ages or (Median)	Female:Male
				
	*prn1*	*prn2*	*prn3*			
1999	3	0	0	3	1,63,46	1:2
2000	1	0	1	2	1,1	2:0
2001	9	0	1	10	(5)	8:2
2002	21	2	3	26	(<6 m)	12:14
2003	4	1	0	5	3 m,11 m,3,2,2	3:2

### Pertactin gene variation

Limited variation was observed in the PRN genes of the 46 Queensland isolates with only three different PRN types detected. Results are presented in Table 1. The *prn1 *allele was identified in 38 of the 46 isolates, *prn2 *in three of the 46 isolates and *prn3 *in five of the 46 isolates. The modified/corrected rapid real-time PCR typing method of Mäkinen et al. [28] correctly identified all 46 of the isolates when compared to full gene sequencing.

From this recent sample it appears that for Queensland *B. pertussis *isolates a PRN type similar to that present in an existing vaccine (*prn1*) has again returned to prevalence (82.5%). Prior to 1989, Poynten et al. reported an Australia wide prevalence of 81% for *prn1 *at a time when a *prn1 *containing Australian WCV had been in use for fifty years [32]. Over the next decade (1989–1998) Poynten et al. subsequently described the emergence of *prn2 *and *prn3*, such that *prn1 *prevalence had dropped to 47% [32]. Based on the results of this study, over the next five years proportions of the variants *prn2 *and particularly *prn3 *in Queensland now appear to have dropped remarkably. Poynten et al. reported the prevalence of *prn3 *in the 22 Queensland isolates from 1989–1998 as 45% (10 of 22), associated with a statistically significant increase Australia wide in the proportion of *B. pertussis *carrying the variant *prn3 *allele between the two time periods, 1967–1985 and 1989–1998 [32]. This collection of 46 isolates from 1999–2003 show that there has now been a statistically significant decrease (P = 0.0034, two sided Fishers exact test) in prevalence of *prn3 *in Queensland compared to 1989–1998, with *prn3 *now accounting for only 11% (5 of 46) of the Queensland total (Table 2).

**Table 2:** Frequency of Queensland *Bordetella pertussis *Sample Pertactin Alleles 1999–2003

*prn *Type	Number	Percentage of Total
1	38	82.5%
2	3	6.5%
3	5	11%

This current observation of a fall in prevalence of PRN variants compared to the PRN type contained in a local vaccine is in marked contrast to that observed in Europe and America where PRN variants have emerged, persisted and significantly replaced isolates possessing a vaccine matching PRN allele [11]. Population studies remain to be done on these isolates to determine if the observation of a re-established dominance of *prn1 *containing vaccine matching strains in Queensland may be a geographic specificity related to a clonal expansion of a distinct *B. pertussis *population. Geographic specificity has been noted in association with populations of *B. pertussis *in both Finland and France [11,24,25,36], and in the persistence of vaccine matching strains in Japan [22,23]. Nonetheless, the observation of a temporal fall in the prevalence of PRN variants and a re-emergence of a PRN allele which is a match to existing vaccines is not consistent with significant vaccination driven change of PRN alleles in the Queensland *B. pertussis *population.

### PT subunit a gene variation

All 46 isolates in this study possessed the A genotype of *ptxS1*. The finding of *ptxS1A *in all of the recent Queensland isolates again shows the predominance of this PT allele in Australian isolates. Despite the historic Australian WCV containing *ptxS1A*, no variation from that phenotype was found by Poynten et al. in 62 isolates tested from the 84 collected between 1967 to 1998 [32]. Although Poynten et al. did report two new variants (*ptxS1F*, *ptxS1G*) derived from *ptxS1A *which contain silent mutations and produced the A type protein [32]. The historical homogeneity of Australian PT alleles is again in marked contrast to northern hemisphere studies that show a change of the dominant PT alleles from *ptxS1B *and *ptxS1D *to *ptxS1A *following the introduction of vaccines with non *ptxS1A *components [9,11,36-39]. Applying the vaccination driven hypothesis it would appear unlikely that *ptxS1 *variants different to *ptxS1A *will emerge, given the Australian WCV (*ptxS1A*) has been replaced by the use of ACVs containing non *ptxS1A *components. Of interest is that no Australian isolate prior to 1967 has been examined and a *ptxS1A *containing WCV was in use from the 1936–45 decade. It may be possible that there was a pre-1967 hereto unrecognised shift in the Australian PT allele prevalence similar to that of the UK. In the UK, the *ptxS1A *allele (as also contained in the UK WCV) began to predominate over *ptxS1B *in clinical isolates from the 1950s and showed 100% prevalence throughout the 1990s [20].

### Sampling

A lack of available cultures of *B. pertussis *from which to perform PRN and PT profiling over the time period compared to total notifications of pertussis disease is a limitation of this study. It is not possible to state that the observed fluctuations in variant prevalence from both studies of Australian isolates to date are free of sampling artefact. A total of 84 isolates (including 22 from Queensland) were collected by Poynten et al. from 1967 through 1998, a period of time where recorded notifications of pertussis for the years 1991 to 1998 alone were 36172 cases [6]. Combining that data and our own, a very small sample of only 130 Australian isolates (68 from Queensland) have been tested from greater than 41407 notifications of pertussis through to 2003. Further, the methods of sampling employed in both studies have been by necessity non-probabilistic, being limited to those cases where an isolate was recovered from a patient and stored. Non-probabilistic sampling by definition does not involve random selection which otherwise allows calculation of probability that the sample is indeed representative of the population it has been drawn from. At best, such convenience sampling may yield good estimates, but no objective evaluation of the precision of sample results can be applied. A co-ordinated attempt in Australia to prospectively obtain clinical isolates from new cases of pertussis is warranted in order to statistically verify these observations. The problem of obtaining cultured isolates in a diagnostic environ rightly shifting to rapid methods such as serology and NAA appears common, and at least one other author has recently recommended active collection of *B. pertussis *strains [24].

## Conclusion

The results of this study compared to historical research on Queensland isolates indicate that *B. pertussis *possessing variant PRN alleles to those in the existing vaccines were less prevalent in Queensland in the five years since the introduction of the Australian ACVs. Whilst the numbers of isolates tested in Australian studies to date consign the data to being a good estimate (at best) rather than being considered statistically valid, an observed return to prevalence of a PRN type identical to that in historical and current local vaccines differs markedly from that seen in many other countries following the introduction of vaccine programs. At present no divergence from the PT *ptxS1A *encoded phenotype has been detected in the limited number of *B. pertussis *strains examined in Australia from 1967 to 2003 despite the historic Australian pertussis WCV containing *ptxS1A *derived proteins. The study data appears to support the view that changes in epidemiology and transmission of pertussis coupled to a shift of the disease from young children to adults/adolescents (and then by association to naive infants) due to waning immunity is the most likely explanation for pertussis re-emergence. Nonetheless, as others have suggested it will continue to remain important to prospectively profile endemic *B. pertussis *for variant genes of the proteins contained in the ACVs [22]. The relative paucity of recovered isolates compared to notified infections, due primarily to non culture based pertussis diagnostics is a confounding factor in the assessment of variant allele prevalence. In the interests of effective long term monitoring of ACV efficacy, for all instances of suspected pertussis infection medical practitioners should also consider submitting samples suitable for culture in addition to requesting rapid diagnostics. Our modifications of the rapid real time PCR method of Mäkinen et al. [28] are currently an acceptable protocol for rapid screening of PRN variants in Australian isolates. We propose to attempt allele typing on DNA extracts from clinical samples which are positive for *B. pertussis *DNA utilising this modified rapid method in order to improve future monitoring in Queensland in the absence of available cultures.

## Competing interests

The author(s) declare that they have no competing interests.

## Authors' contributions

SB initiated the study collaborative; ATS and SB performed the laboratory work and contributed equally to the final version of the manuscript.

## Pre-publication history

The pre-publication history for this paper can be accessed here:



## References

[B1] (2003). The Australian Immunisation Handbook.

[B2] McIntyre P, Amin J, Gidding H, Hull B, Torvaldsen S, Tucker A, Turnbull F, Burgess M (2000). Vaccine preventable diseases and vaccination coverage in Australia, 1993-1998. Commun Dis Intell.

[B3] (2003). Vaccine-preventable diseases and vaccination coverage in Australia, 1999-2000. N S W Public Health Bull.

[B4] Brotherton J, McIntyre P, Puech M, Wang H, Gidding H, Hull B, Lawrence G, MacIntyre R, Wood N, Armstrong D (2004). Vaccine preventable diseases and vaccination coverage in Australia 2001 to 2002. Commun Dis Intell.

[B5] Miller M, Roche P, Yohannes K, Spencer J, Bartlett M, Brotherton J, Hutchinson J, Kirk M, McDonald A, Vadjic C (2005). Australia's notifiable diseases status, 2003: Annual report of the National Notifiable Diseases Surveillance System. Commun Dis Intell.

[B6] National Notifiable Diseases Surveillance System. http://www1.health.gov.au/cda/Source/Rpt_4_sel.cfm.

[B7] de Melker HE, Schellekens JF, Neppelenbroek SE, Mooi FR, Rumke HC, Conyn-van Spaendonck MA (2000). Reemergence of pertussis in the highly vaccinated population of the Netherlands: observations on surveillance data. Emerg Infect Dis.

[B8] Guris D, Strebel PM, Bardenheier B, Brennan M, Tachdjian R, Finch E, Wharton M, Livengood JR (1999). Changing epidemiology of pertussis in the United States: increasing reported incidence among adolescents and adults, 1990-1996. Clin Infect Dis.

[B9] Mooi FR, van Loo IH, King AJ (2001). Adaptation of Bordetella pertussis to vaccination: a cause for its reemergence?. Emerg Infect Dis.

[B10] Bass JW, Wittler RR (1994). Return of epidemic pertussis in the United States. Pediatr Infect Dis J.

[B11] Godfroid F, Denoel P, Poolman J (2005). Are vaccination programs and isolate polymorphism linked to pertussis re-emergence?. Expert Rev Vaccines.

[B12] von Konig CH, Halperin S, Riffelmann M, Guiso N (2002). Pertussis of adults and infants. Lancet Infect Dis.

[B13] Andrews R, Herceg A, Roberts C (1997). Pertussis notifications in Australia, 1991 to 1997. Commun Dis Intell.

[B14] Forsyth KD, Campins-Marti M, Caro J, Cherry JD, Greenberg D, Guiso N, Heininger U, Schellekens J, Tan T, von Konig CH, Plotkin S (2004). New pertussis vaccination strategies beyond infancy: recommendations by the global pertussis initiative. Clin Infect Dis.

[B15] Forsyth K, Tan T, von Konig CH, Caro JJ, Plotkin S (2005). Potential strategies to reduce the burden of pertussis. Pediatr Infect Dis J.

[B16] Forsyth K, Nagai M, Lepetic A, Trindade E (2005). Pertussis immunization in the global pertussis initiative international region: recommended strategies and implementation considerations. Pediatr Infect Dis J.

[B17] Poynten M, Hanlon M, Irwig L, Gilbert GL (2002). Serological diagnosis of pertussis: evaluation of IgA against whole cell and specific Bordetella pertussis antigens as markers of recent infection. Epidemiol Infect.

[B18] Notifiable Diseases Report 1997-2001. http://www.health.qld.gov.au/phs/Documents/cdu/15896.pdf.

[B19] Mooi FR, van Oirschot H, Heuvelman K, van der Heide HG, Gaastra W, Willems RJ (1998). Polymorphism in the Bordetella pertussis virulence factors P.69/pertactin and pertussis toxin in The Netherlands: temporal trends and evidence for vaccine-driven evolution. Infect Immun.

[B20] Fry NK, Neal S, Harrison TG, Miller E, Matthews R, George RC (2001). Genotypic variation in the Bordetella pertussis virulence factors pertactin and pertussis toxin in historical and recent clinical isolates in the United Kingdom. Infect Immun.

[B21] Denoel P, Godfroid F, Guiso N, Hallander H, Poolman J (2005). Comparison of acellular pertussis vaccines-induced immunity against infection due to Bordetella pertussis variant isolates in a mouse model. Vaccine.

[B22] Guiso N, Boursaux-Eude C, Weber C, Hausman SZ, Sato H, Iwaki M, Kamachi K, Konda T, Burns DL (2001). Analysis of Bordetella pertussis isolates collected in Japan before and after introduction of acellular pertussis vaccines. Vaccine.

[B23] Kodama A, Kamachi K, Horiuchi Y, Konda T, Arakawa Y (2004). Antigenic divergence suggested by correlation between antigenic variation and pulsed-field gel electrophoresis profiles of Bordetella pertussis isolates in Japan. J Clin Microbiol.

[B24] van Amersfoorth SC, Schouls LM, van der Heide HG, Advani A, Hallander HO, Bondeson K, von Konig CH, Riffelmann M, Vahrenholz C, Guiso N, Caro V, Njamkepo E, He Q, Mertsola J, Mooi FR (2005). Analysis of Bordetella pertussis populations in European countries with different vaccination policies. J Clin Microbiol.

[B25] Caro V, Njamkepo E, Van Amersfoorth SC, Mooi FR, Advani A, Hallander HO, He Q, Mertsola J, Riffelmann M, Vahrenholz C, Von Konig CH, Guiso N (2005). Pulsed-field gel electrophoresis analysis of Bordetella pertussis populations in various European countries with different vaccine policies. Microbes Infect.

[B26] Muyldermans G, Pierard D, Hoebrekx N, Advani R, Van Amersfoorth S, De Schutter I, Soetens O, Eeckhout L, Malfroot A, Lauwers S (2004). Simple Algorithm for Identification of Bordetella pertussis Pertactin Gene Variants. J Clin Microbiol.

[B27] Fiett J, Letowska I, Gniadkowski M, Hryniewicz W (2003). The new strategy for allele identification of the genes coding for pertussis toxin subunit S1 (ptx S1) and pertactin (prn) in Bordetella pertussis. J Microbiol Methods.

[B28] Makinen J, Viljanen MK, Mertsola J, Arvilommi H, He Q (2001). Rapid identification of Bordetella pertussis pertactin gene variants using LightCycler real-time polymerase chain reaction combined with melting curve analysis and gel electrophoresis. Emerg Infect Dis.

[B29] Tamura M, Nogimori K, Murai S, Yajima M, Ito K, Katada T, Ui M, Ishii S (1982). Subunit structure of islet-activating protein, pertussis toxin, in conformity with the A-B model. Biochemistry.

[B30] Sekura RD, Fish F, Manclark CR, Meade B, Zhang YL (1983). Pertussis toxin. Affinity purification of a new ADP-ribosyltransferase. J Biol Chem.

[B31] Makinen J, Mertsola J, Viljanen MK, Arvilommi H, He Q (2002). Rapid typing of Bordetella pertussis pertussis toxin gene variants by LightCycler real-time PCR and fluorescence resonance energy transfer hybridization probe melting curve analysis. J Clin Microbiol.

[B32] Poynten M, McIntyre PB, Mooi FR, Heuvelman KJ, Gilbert GL (2004). Temporal trends in circulating Bordetella pertussis strains in Australia. Epidemiol Infect.

[B33] Mooi FR, Hallander H, Wirsing von Konig CH, Hoet B, Guiso N (2000). Epidemiological typing of Bordetella pertussis isolates: recommendations for a standard methodology. Eur J Clin Microbiol Infect Dis.

[B34] Strike PW (1991). Statistical Methods in Laboratory Medicine.

[B35] Greenberg DP, von Konig CH, Heininger U (2005). Health burden of pertussis in infants and children. Pediatr Infect Dis J.

[B36] Weber C, Boursaux-Eude C, Coralie G, Caro V, Guiso N (2001). Polymorphism of Bordetella pertussis isolates circulating for the last 10 years in France, where a single effective whole-cell vaccine has been used for more than 30 years. J Clin Microbiol.

[B37] Mooi FR, He Q, van Oirschot H, Mertsola J (1999). Variation in the Bordetella pertussis virulence factors pertussis toxin and pertactin in vaccine strains and clinical isolates in Finland. Infect Immun.

[B38] Kourova N, Caro V, Weber C, Thiberge S, Chuprinina R, Tseneva G, Guiso N (2003). Comparison of the Bordetella pertussis and Bordetella parapertussis isolates circulating in Saint Petersburg between 1998 and 2000 with Russian vaccine strains. J Clin Microbiol.

[B39] Cassiday P, Sanden G, Heuvelman K, Mooi F, Bisgard KM, Popovic T (2000). Polymorphism in Bordetella pertussis pertactin and pertussis toxin virulence factors in the United States, 1935-1999. J Infect Dis.

